# LKB1 Regulates Cerebellar Development by Controlling Sonic Hedgehog-mediated Granule Cell Precursor Proliferation and Granule Cell Migration

**DOI:** 10.1038/srep16232

**Published:** 2015-11-09

**Authors:** Yuqin Men, Aizhen Zhang, Haixiang Li, Yecheng Jin, Xiaoyang Sun, Huashun Li, Jiangang Gao

**Affiliations:** 1 School of Life Science and Key Laboratory of the Ministry of Education for Experimental Teratology, Shandong University, Jinan 250100, China; 2 SARITEX Center for Stem Cell, Engineering Translational Medicine, Shanghai East Hospital, Advanced Institute of Translational Medicine, Tongji University School of Medicine, Shanghai 200123, China; 3 Center for Stem Cell&Nano-Medicine, Shanghai Advanced Research Institute, Chinese Academy of Sciences, Shanghai 200123, China; 4 Shenzhen Key Laboratory for Molecular Biology of Neural Development, Shenzhen Institutes of Advanced Technology, Chinese Academy of Sciences, Shenzhen 518000, Guangdong, China

## Abstract

The Liver Kinase B1 (LKB1) gene plays crucial roles in cell differentiation, proliferation and the establishment of cell polarity. We created LKB1 conditional knockout mice (LKB1^Atoh1^ CKO) to investigate the function of LKB1 in cerebellar development. The LKB1^Atoh1^ CKO mice displayed motor dysfunction. In the LKB1^Atoh1^ CKO cerebellum, the overall structure had a larger volume and morelobules. LKB1 inactivationled to an increased proliferation of granule cell precursors (GCPs), aberrant granule cell migration and overproduction of unipolar brush cells. To investigate the mechanism underlying the abnormal foliation, we examined sonic hedgehog signalling (Shh) by testing its transcriptional mediators, the Gli proteins, which regulate the GCPs proliferation and cerebellar foliation during cerebellar development. The expression levels of Gli genes were significantly increased in the mutant cerebellum. *In vitro* assays showed that the proliferation of cultured GCPs from mutant cerebellum significantly increased, whereas the proliferation of mutant GCPs significantly decreased in the presence of a Shh inhibitor GDC-0049. Thus, LKB1 deficiency in the LKB1^Atoh1^ CKO mice enhanced Shh signalling, leading to the excessive GCP proliferation and the formation of extra lobules. We proposed that LKB1 regulates cerebellar development by controlling GCPs proliferation through Shh signalling during cerebellar development.

The cerebellum is a critical motor organ that controls both motor coordination and motor learning^1^ and also plays a critical role in cognition, affect and behaviour. The growth and foliation of the cerebellum is a distinct process in cerebellar morphogenesis during development. The cerebellar cortex is divided into three distinct cellular layers in the adult: the molecular layer (ML), the Purkinje cell layer (PCL), and the inner granule cell layer (ICL)^2^. The most superficial ML contains Purkinje cell (PC) dendrites, granule cell (GC) axons, stellate and basket cell interneurons and Bergmann glia^1^,^3^,^4^,^5^. The single, middle PCL is comprised of the somata of both PCs and Bergmann glia^6^. The innermost IGL primarily consists of the most numerous neuronal cell type of the brain, GCs, and the somata of Golgi cells and unipolar brush cells (UBCs)^2^.

The formation of the cerebellum spans embryonic and postnatal development, which initiates at embryonic day 9 (E9) and matures at approximately postnatal day 16 (P16) in mice^7^,^8^,^9^. Two primary regions are known to give rise to the neurons that make up the cerebellum. The first region is the ventricular zone in the fourth ventricle, and this region produces PCs, Golgi cells, basket cells, stellate cells, and small, deep cerebellar nuclei neurons^1^,^5^. The second region is the rhombic lip (RL). Cerebellar granule cells precursors (GCPs) are generated in the RL region and migrate to the outer pial surface of the RL at approximately E12.5, forming the external granular layer (EGL)^10^. After birth, the GCPs in the EGL continue to proliferate, differentiate, migrate and form the internal granular layer (IGL)^1^,^10^. Each of these steps must be coordinated for cerebellar development. However, the molecular mechanisms that regulate these processes are not fully understood.

The LKB1 gene is an important serine/threonine kinase11 (STK11). LKB1 encodes a 48-kDa protein, which is localized in the nucleus^11^ and translocated to the cytoplasm upon activation^11^,^12^. LKB1 is ubiquitously expressed in various tissues, particularly in the brain, hippocampus, liver, testes and skeletal muscles, and it plays crucial roles in cell differentiation, proliferation, migration, apoptosis, the DNA damage response and differentiation. Based on the wide expression and significant roles of the LKB1 gene, conventional LKB1 knockout mice are embryonic lethal at E8-9^13^,^14^. The LKB1 conventional knockout mice displayed a variety of developmental abnormalities, particularly in angiogenesis and the nervous system^13^,^14^. Some studies have been reported functions of LKB1 in the nervous system using conditional knockouts. Cortex-specific LKB1 deletion using Emx-Cre mice showed abnormal axon specification in cerebral cortex of developing mice^15^. LKB1 conditional knockout mice using the pancreatic and hypothalamic Rip2-Cre developed hind-limb paralysis and axon degeneration in spinal cord neurons^16^. LKB1 deletion using Ubi-Cre and Nestin-CreER^T2^ resulted in the failure to establish axon-dendrite polarity during dendrite morphogenesis in adult hippocampal neurons during neogenesis^17^. NEX-Cre-mediated LKB1 deficiency in cortical pyramidal neurons showed that LKB1 is important in regulating axon terminal branching^18^. Thus, LKB1 plays essential roles in ensuring the normal development of the nervous system.

As mentioned above, the wide expression and critical functions of LKB1 were demonstrated in the nervous system in mice. However, there are currently no reported studies on the role of LKB1 during cerebellar development. We undertook a pretest and detected strong LKB1 expression in the cerebellum. To investigate the role of LKB1 in cerebellar development, we created cerebellum-specific LKB1 conditional knockout mice by crossing LKB1^LoxP/LoxP^ mice with Atoh1-Cre mice. In our study, we determined that the LKB1-deficient mice showed motor dysfunction and cerebellar malformation, including a larger volume and extra lobules in the mutant cerebellum. We also found abnormal proliferation of the GCPs and the failure of GC migration in the LKB1^Atoh1^ CKO mice. Thus, we propose that LKB1 may play an important role in cerebellar development.

## Materials and Methods

### Animals

Mice homozygousfor floxed LKB1 mice (LKB1^LoxP/LoxP^)^19^ were crossed with Atoh1-Cre mice^20^. Atoh1-Cre activities were confined to the developing the hindbrain, the spinal cord, inner ear and the intestine. In the cerebellum, Atoh1-Cre activities were detected in the granule cell precursors in the developing rhombic lip (RL). Thus, the Atoh1-Cre mouse line usually serves as a tool for the functional study of genes in the cerebellum.

We genotyped the wild-type mice, heterozygous mice and homozygous knockout mice by PCRs. DNA was extracted from mice tail snips for PCR analysis. Mice were genotyped for Cre using the following primers: Cre-F (5′-AGCTAAACATGCTTCATCGTCGGTC-3′) and Cre-R (5′-TATCCAGGTTACGGATATAGTTCATG-3′). The PCR genotyping of the floxed LKB1 allele was detectedby the following primers: LKB1-F (5′-ATCGGAATGTGATCCAGCTT-3′) and LKB1-R (5′-GAGATGGGTACCAGGAGTTGGGGCT-3′).

All animal experimental procedures were approved by Ethics Committee of Shandong University. Animal management was performed strictly in accordance with the standards of the Animal Ethics Committee of Shandong University.

### Histology and immunohistochemistry analysis

The mice were perfused with 4% paraformaldehyde, and then the cerebella were dissected from the heads and post-fixed in the same fixative overnight at 4 °C. For paraffin sections, the cerebellum was dehydrated via an ethanol series from 30% to 100%. The cerebellum was clarified with xylene, embedded in paraffin, sectioned at 10 μm thickness and stained using haematoxylin and eosin staining for morphological analysis. For frozen section immunostaining, the cerebellum samples were equilibrated for 4 hours in 15% sucrose at room temperature and later in 30% sucrose overnight at 4 °C. Finally, the samples were embedded in OCT compound and frozen at −20 °C followed by liquid nitrogen. The tissue blocks were sectioned at 10 μm thickness. Sections were washed with 10 mM PBS and blocked (10% goat serum) for 30 min at room temperature. Primary antibodies were diluted in 10 mM PBS, and the samples were incubated with primary antibodies overnight at 4 °C. After three wash in PBS, samples were washed with PBS and incubated at room temperature for 1 h in secondary antibodies (goat anti-rabbit Alexa-488 or Alexa-568, 1:400; Invitrogen) diluted in the10 mM PBS. Samples were then stained with nuclear stain (DAPI) were applied to the samples for 15 min at room temperature followed by a final PBS wash. Cerebellar sections were imaged with a LSM 700 confocal microscope. Primary antibodies included anti-LKB1 (1:200, Upstate), anti-BrdU (1:400, Sigma), anti-PH3 (1:400, Bioworlde), anti-NeuN (1:200, Abcam), anti-NeuN (1:500, Cell Signaling Technology), anti-GFAP (1:200, Cell Signaling Technology), anti-calbindinD28K (1:1000, Abcam), anti-Cleaved Caspase3 (1:400, CST), anti-Tbr2 (1:200, Abcam) and anti-cyclinD1 (1:500, Abcam).

### Behaviour tests

To understand the underlying pathophysiology of cerebella in the LKB1^Atoh1^ CKO mice, three tests were performed to measure motor coordination, including gait test, rotarod test, and tightrope test. The gait test was performed as previously described^21^. Nontoxic black ink was applied to the hind paws of each mouse. Mice were allowed to walk through a long corridor (25 cm × 10 cm × 9 cm) on white paper. This activity allows multiple gait cycles of the wild-type and LKB1^Atoh1^ CKO mice. Gait width was determined by measuring the perpendicular distance of a given step to its opposite preceding and succeeding steps. The rotarod test was performed as previously described^22^. Mice were tested for five consecutive days using the rotarod apparatus with a speed of 10 rpm. The apparatus consisted of a gridded plastic roller (3 cm in diameter and 5.5 cm in length) that was flanked by two round plates (30 cm in diameter) to prevent the animals from escaping. The time taken for each mouse to maintain balance on the rotarod was measured. The latency to fall from the rod was recorded and the maximum time allowed on the rotarod was 15 min. Tightrope test was performed as previously described^23^. Briefly, the rope (2 mm in diameter and 50 cm in length) was marked by black maker pen at 5-cm intervals, and the tank (50 cm in diameter and 30 cm in height) was filled with water to a depth of 10 cm. The suspension time the mice were on the rope until they fall into the water was recorded and the maximum time of suspension allowed on the rope was 60 s. Horizontal movement was measured by recording the number of marks crossed on the rope. Suspension time and number of marks crossed were averaged for each mouse. A final transformed score was calculated for each mouse: Transformed Score = suspension time + 10 × the number of crossings. A lower transformed score indicates inferior performance motor coordination.

### Cerebellar granule cell precursors culture and proliferation analysis

Cerebella from P7 animals were minced into small pieces using scissors and were digested at 37 °C for 30 min in 2 mg/ml papain (Solarbio) and 10 μg/ml DNase (Sigma). Single-cell suspension was obtained via trituration with glass pipettes and then centrifuged through a 35%–65% Percoll gradient (Solarbio). GCPs were harvested from the 35%–65% interface and centrifuged at 2000 rpm for 5 min at room temperature. Cells in high glucose DMEM medium (HyClone) were washed and suspended in the GCP culture medium, which is composed of Neurobasal A medium (Invitrogen) with 2 mM L-glutamine, 2% B27 supplement, and penicillin/streptomycin. GCPs were plated on poly-D-lysine (100 μg/ml)-coated dishes. Cultures were maintained in 5% CO_2_ at 37 °C and analyzed 24 h after incubation. For *vitro* proliferation assays, GCP cells were treated with 1 μM Shh inhibitor Vismodegib (GDC-0449, Selleckchem) in DMSO or DMSO alone as control for 48 h, and then 10 μM BrdU for 2 h. For immunostaining analysis, GCPs in culture were immunostained with antibodies against BrdU and NeuN. The relative rate of BrdU^+^ cells among NeuN^+^ cells were quantified to examine GCP proliferation *in vitro*.

### BrdU labelling

To quantitate granule cell precursor proliferation, the mice were injected with BrdU (50 μg/g) at P3.5. To quantitate granule cell migration, the mice were injected with BrdU (50 μg/g) at P7 and analysed 24 h and 96 h after BrdU-injection. The cerebella were dissected from the heads of mice and fixed with 4% paraformaldehyde. The samples were embedded in OCT and sectioned at 10 μm. BrdU detection was performed according to the manufacturer’s instructions (Sigma). All data are presented as the means ± SEM, and the data were analysed via Student’s t-test. The significance level was set at P < 0.05 for all statistical analyses.

### Western blot

Cerebellum from the controls and LKB1^Atoh1^ CKO mice were incubated in cell lysis buffer (10 mM Tris, pH = 7.4, 1% Triton X-100, 150 mM NaCl, 1 mM EDTA, 0.2 mM PMSF) and the proteins were extracted^24^. The proteins from the samples (20 μg) were subjected to SDS-polyacrylamide gel electrophoresis and blotted onto a polyvinylidene difluoride membrane (PVDF). Western blots were performed as described previously. The following primary antibodies were used: anti-LKB1 (1:500, Bioworlde), anti-Gli1 (1:2000, Bioss), anti-Gli2 (1:1000, RD), anti-Gli3 (1:1000, Proteintech), anti-AMPK (1:500, Cell Signaling Technology)anti-p-AMPK (1:500, Cell Signaling Technology), anti-β-actin (1:5000, Bioworlde), anti-Tbr2 (1:1000, Abcam) and anti-cyclinD1 (1:5000, Abcam). All data are presented as means ± SEM and data analyses were analyzed via Student’s t-test. A significance level was set to P < 0.05 for all statistical analyses.

## Results

### Conditional inactivation of LKB1 in the developing cerebellum

**Figure 1:**
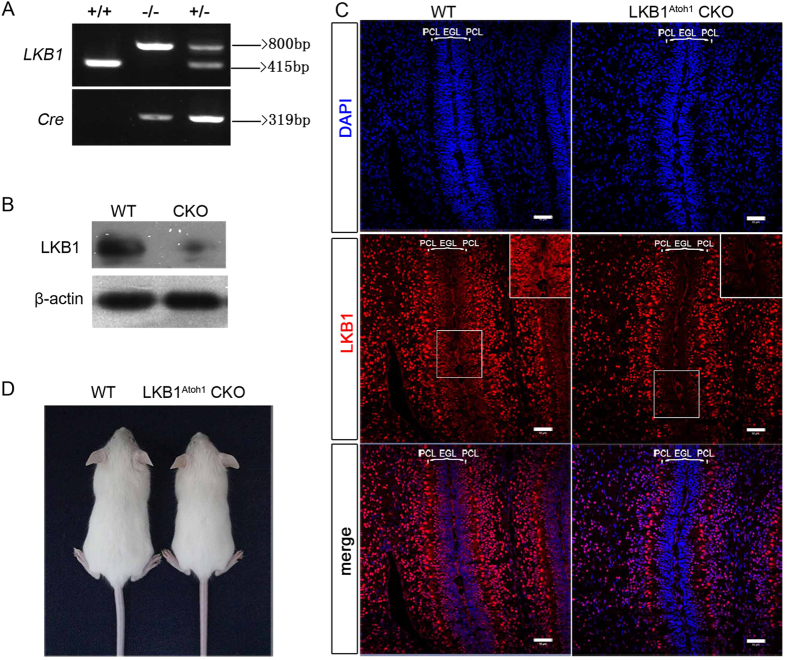
Generation of LKB1^Atoh1^ CKO mice. (**A**) Mouse genotyping by PCR analysis. Lanes: wild-type (+/+), homozygous (−/−) and heterozygous (+/−) mice. (**B**) Western blot of Lkb1 in the cerebellum of P14 LKB1^Atoh1^ CKO and wild-type mice. A 48.6-kD LKB1 protein was obviously decreased in the cerebellum of LKB1^Atoh1^ CKO mice (CKO) compared to the controls (WT). (**C**) Expression pattern of LKB1 in the P3.5 cerebellum. Confocal images of the cerebellum in wild-type and LKB1^Atoh1^ CKO mice. In the P3.5 wild-type cerebellum, LKB1 was primarily localized throughout the entire lobe, including the PCL and EGL (parentheses) which mainly consist of the granule cell precursors (GCPs), while in the LKB1^Atoh1^ CKO cerebellum, LKB1 expression was not detected in the GCPs of the ECL. Inserts represent high magnification view of the boxed areas. Highly magnified images confirmed no detectable expression in the GCPs of the EGL in mutant mice. Red, LKB1; Blue, DAPI. Scale bar: 50 μm. (**D**) Gross morphology of P21 wild-type and LKB1^Atoh1^ CKO mice. There were no obvious differences, except for the smaller body size of the mutant mice compared to the controls.

We utilized an *Atoh1-Cre* line to inactivate LKB1 in granule cell precursors from the developing rhombic lip (RL) that give rise to both granule cells and unipolar brush cells (UBCs) in the cerebellum^3^,^25^. LKB1 conditional knockout mice (LKB1^Atoh1^ CKO mice) were obtained and genotyped by PCR analysis (Fig. 1A). The amount of LKB1 protein was measured in the whole cerebellum using Western blots, and the results showed an obvious decrease of LKB1 protein in the LKB1^Atoh1^ CKO mice compared with the wild-type mice (Fig. 1B). We examined the expression of LKB1 in the P3.5 cerebellum of the wild-type mice and LKB1^Atoh1^ CKO mice using immunofluorescence. LKB1 showed a high level of expression in the multiple cerebellar cell types in the wild type mice, including the GCs and PCs, while there was no LKB1 was detected in GCPs in the EGL of the LKB1^Atoh1^ CKO cerebellum (Fig. 1C).

### LKB1^Atoh1^ CKO mice exhibit motor coordination impairments

**Figure 2:**
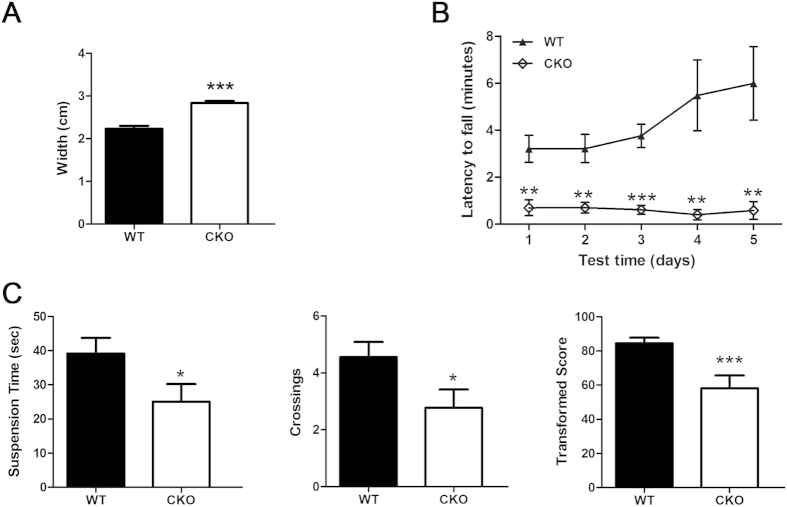
The behaviour tests in P30 LKB1^Atoh1^ CKO and wild-type mice. (**A**) Footprint assay in gait test in wild-type mice and LKB1^Atoh1^ CKO mice. The stride width (cm) of hindpaw footprint was significantly wider in the mutant mice compared with wild-type mice (P = 5 × 10 ^−7^). (**B**) Rotarod tests were performed on wild-type and LKB1^Atoh1^ CKO mice for 5 consecutive days using a rotarod treadmill. The duration time for the mice to stay on the rotary rod at 10 rpm was counted and compared. The mutant mice exhibited impaired motor coordination with a shorter duration time compared to the wild-type mice (P = 0.0036; 0.0028; 0.00014; 0.0074; 0.0072). (**C**) Tightrope suspension time (P = 0.042) and crossing (P = 0.049) were measured in three trials in tightrope test. Transformed scores (P = 8.2 × 10 ^−4^) were calculated. Results showed that transformed score was significantly lower in the mutant mice than those of wild-type mice. The error bars indicate the SEM. *P < 0.05; **P < 0.01; ***P < 0.001 compared to the WT by Student’s t-test; n = 6 animals for each group.

In contrast to the LKB1 null mice that died on embryonic day 8–9^13^, our LKB1^Atoh1^ CKO mice survived to adulthood. The LKB1^Atoh1^ CKO mice were fertile and showed no other obvious abnormalities in their gross morphology (Fig. 1D) except for reduced body weight from postnatal day 14 to adulthood (data not shown). The cerebellum is responsible for the regulation of muscle tone, smooth coordination of somatic motor, and influence and maintenance of equilibrium^26^. We examined the motor coordination in LKB1^Atoh1^ CKO mice using gait test, rotating rod task, and tightrope test. The walking pattern was examined in the gait test. There was a significant difference was observed when comparing step width. The stride width of hindpaw footprint was significantly wider in the mutant mice compared with wild-type mice (Fig. 2A). Rotarod tests showed that the mutant mice were much slower in learning how to balance themselves on the rod, and the retention time of LKB1-deficient mice on the rod was much shorter than wild-type mice (Fig. 2B). In the tightrope test, the suspension time of the mutant mice was shorter than the wild-type controls. Transformed scores were calculated and LKB1^Atoh1^ CKO mice showed lower transformed score, indicating their inferior performance in this test (Fig. 2C). These results revealed the deficient performance in the motor coordination in the LKB1^Atoh1^ CKO mice compared with the control mice.

### Abnormal cerebellar development in LKB1-deficient mice

**Figure 3:**
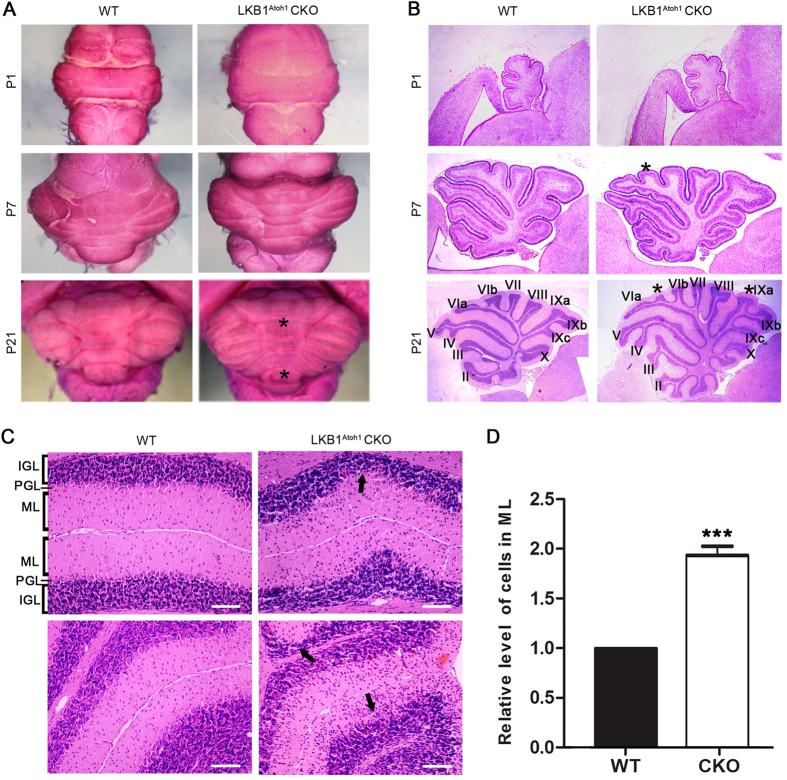
Morphological abnormalities of the cerebellum in the mutant mice. (**A**) Abnormal volume of the cerebella in the LKB1^Atoh1^ CKO mice. At P1 and P7, the volume of the LKB1^Atoh1^ CKO cerebellum was similar to the wild-type. However, the P21 mutant cerebellum was obviously larger with more lobules (asterisk) compared to the wild-type mice. (**B**) Histological sections of the cerebellar vermis in the wild-type and LKB1^Atoh1^ CKO mice. At P1, the mutant cerebellum possessed the five typical cardinal lobes, which were similar to the wild-type cerebellum. At P7, the wild-type cerebellum exhibited well-defined lobules. However an additional lobule (asterisk) was observed in the mutant cerebellum, compared to the wild type cerebellum. At P21, the mutant cerebellum was larger with more lobules (asterisk) compared to the wild-type. (**C**) Cerebellar paraffin sections were stained with HE and the results showed a darkly stained IGL (parentheses) and PCs and a lightly stained ML (parentheses) at high magnification. At P21, the number of cells in the ML (framed) was significantly increased, with a concomitant reduction of the cells in the IGL (arrows) in the LKB1^Atoh1^ CKO mice compared to the wild-type mice. Scale bar: 100 μm. (**C’**) Quantitative analysis of the relative number of cells in the ML (P = 6 × 10 ^−6^). A significant increase in the number of cells in the ML was found in the LKB1^Atoh1^ CKO cerebellum, compared to the wild-type cerebellum. The error bars indicate the SEM. *P < 0.05; **P < 0.01; ***P < 0.001 compared to the WT by Student’s t-test; n = 4 animals for each group.

During early postnatal development, we found that the cerebellar volume of the LKB1^Atoh1^ CKO mice is similar to the controls before P7 (Fig. 3A). In the adult mutant mice, the overall structure of the cerebellum was abnormal, with an obviously larger volume and more lobules at P21 (Fig. 3A). To examine the cerebellar structures in the LKB1^Atoh1^ CKO mice in detail, we examined paraffin sections of the cerebellum by HE staining. The results showed that the mutant cerebellum had a similar morphology and histological structure to the controls at P1, which both had the five typical cardinal lobes formed by four principal fissures (Fig. 3B). At P7, the cardinal lobes had already emerged in the cerebellum of wild-type mice, while an additional lobe began to be formed between the sixth and seventh main lobes in the cerebellum of LKB1^Atoh1^ CKO mice (Fig. 3B). After P21, more additional fissures successively divided the cardinal lobes into lobules in the sagittal cerebellar sections of LKB1^Atoh1^ CKO mice compared with the controls (Fig. 3B). All LKB1^Atoh1^ CKO mice were found to have additional lobes in cerebella.

**Figure 4:**
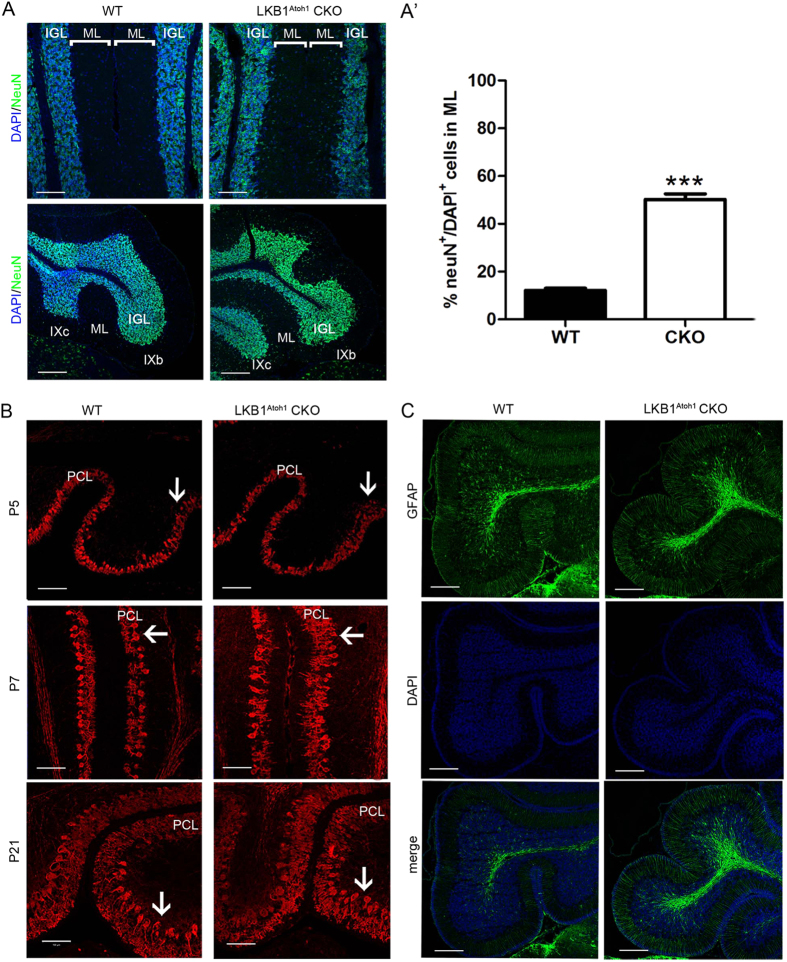
Abnormal granule cells and Purkinje cells in the cerebellum of LKB1^Atoh1^ CKO mice. (**A**) The cerebella of P30 wild-type and LKB1^Atoh1^ CKO mice were stained with NeuN, a GC marker. The LKB1^Atoh1^ CKO cerebellum showed an abnormal accumulation of GCs in the ML compared with the wild-type cerebellum. Green, NeuN; Blue, DAPI. Scale bars: 100 μm. (**A’**) Quantification analysis of NeuN^+^ cells confirmed an abnormal accumulation of GCs in the ML of mutant cerebellum compared to the wild-type cerebellum (P = 2 × 10 ^−6^). The error bars indicate the SEM. *P < 0.05; **P < 0.01; ***P < 0.001 compared to the WT by Student’s t-test; n = 4 for each group. (**B**) Aberrant PC development in LKB1^Atoh1^ CKO mice. CalbindinD28K immunostaining, a marker of Purkinje cells, showed that PCs in the P5 mutant cerebellum were clearly accumulated (arrows) in some parts of the mutant cerebellum compared to the wild-type cerebellum. At P7, the PCs were accumulated (arrows) and showed scant dendritic arborisation (arrows) in the LKB1^Atoh1^ CKO cerebellum, while the PCs of control cerebellum were present in a single layer. At P21, in contrast to the PCs of the wild-type cerebellum, some of the PCs in the mutant cerebellum were accumulated (arrows) in more than one layer and were disorganised (arrows). Red, CalbindinD28K; Blue, DAPI. Scale bar: 100 μm. (**C**) Cerebellar section of the P30 wild-type and LKB1^Atoh1^ CKO mice were stained using the anti-GFAP antibody, a marker of Bergmann glia. No discernible developmental defects were observed in the BGs of the mutant cerebellum compared to the controls. Green, GFAP; Blue, DAPI. Scale bars, 100 μm.

We further examined the structure of the cerebellar lamina in detail using high magnification images of sagittal sections from the P21 cerebellum. Three distinct laminar layers, the ML, PCL and IGL, were observed in the wild type cerebellum, and the boundary of the three layers in the LKB1^Atoh1^ CKO cerebellum was also clearly defined (Fig. 3C). However, in contrast to the controls, the cell number in the ML was dramatically increased, with a concomitant reduction in some parts of the IGL in the LKB1^Atoh1^ CKO cerebellum (Fig. 3C,D). We also examined the distribution of granule cells in the ML of the mutant cerebellum at P30 (Fig. 4A). By immunostaining with NeuN, a marker for GCs, we observed a large number of GCs in the mutant ML, while almost no granule cells were found in the ML of the control mice (Fig. 4A,A’). This revealed that granule cells accumulated and persisted in the ML of the mutant cerebellum.

PCs are another critical component of the cytoarchitecture of the cerebellar cortex and exhibit an orderly monolayer alignment. The development of the PCs dendritic trees depends on the proper formation of parallel fibres, the GC axons^27^. Previous studies reported that granule neuron defects could cause abnormal Purkinje cell maturation^28^. To determine whether the PCs were affected by the abnormal GC development in our mutant mice, we stained the Purkinje cells at P5, P7 and P21 with calbindinD28K, a PC marker. We observed a disorganized PCL at different stages (Fig. 4B). The patterning of the Purkinje cells was disrupted, and they failed to form the Purkinje cell monolayer in the mutant cerebellum (Fig. 4B). At P5, the cerebellum of the control mice had well-organized PCs, while clustered PCs were observed in the mutant cerebellum (Fig. 4B). At P7, we found stunted dendritic branches in the mutant cerebellum compared to the wild-type cerebellum (Fig. 4B). At P21, the PCs were well-organized in a single cell layer in the controls, while some of PCs were disorganized in the PCL and aligned to multiple cell layers in the LKB1^Atoh1^ CKO cerebellum (Fig. 4B). Thus, the PC development is aberrant in the LKB1^Atoh1^ CKO mice.

We next examined the morphology of the Bergman glial cells (BGs), an essential glial structure for granule cell migration during development^1^. BGs were located either immediately below or interspersed between the Purkinje cell bodies, forming a Purkinje cell and BG (PC/BG) monolayer^5^. Using GFAP immunofluorescence, a marker for the BG, we found that the Bergmann glia structure appeared morphologically normal in the LKB1^Atoh1^ CKO cerebellum (Fig. 4C).

### LKB1 inactivation increases GCP proliferation but does not affect GCP apoptosis in the LKB1^Atoh1^ CKO mice

**Figure 5:**
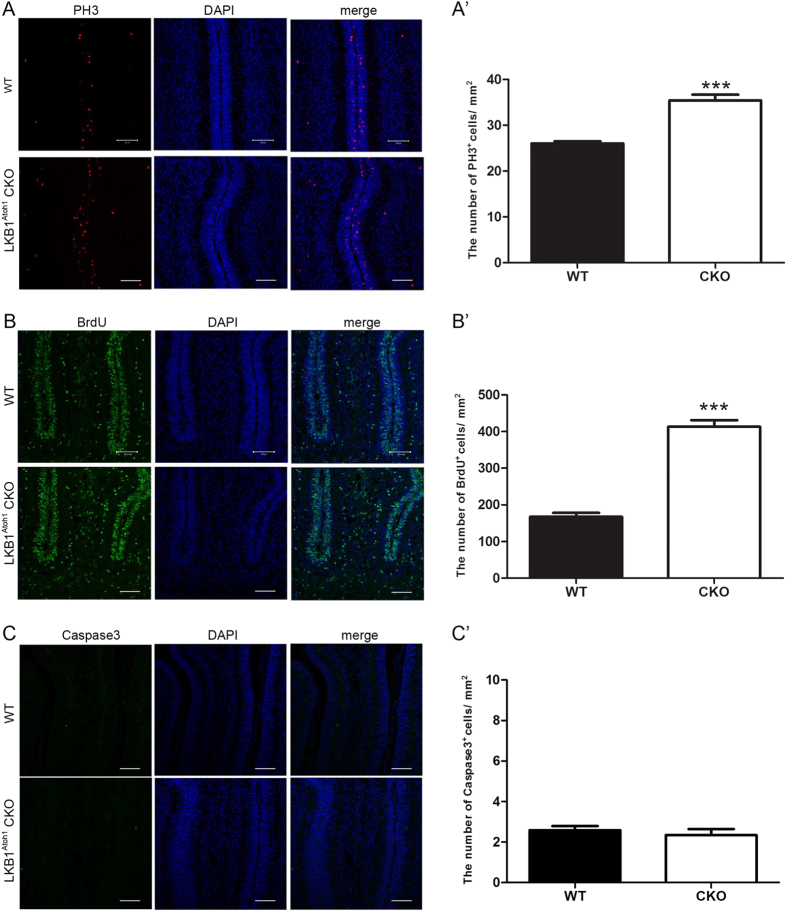
Analysis of GCP proliferation and opoptosis in the LKB1^Atoh1^ CKO mice. (**A**) Sections of P3.5 wild-type and LKB1^Atoh1^ CKO cerebellum were stained with the anti-PH3 antibody. More PH3^+^ cells were observed in the mutant cerebellum compared to the wild-type cerebellum, indicating increased GCP proliferation. Red, PH3; Blue, DAPI. Scale bars: 100 μm. (**A’**) The number of the PH3^+^ cells per unit size of sagittal sections from the entire cerebellum was quantified in ImageJ (P = 1 × 10 ^−4^). (**B**) Immunofluorescence of the BrdU pulse (2 hours) in frozen sections of the P3.5 cerebellum. The cerebellar sections were stained with an anti-BrdU antibody, and more BrdU^+^ cells were observed in the mutant cerebellum compared to the wild-type cerebellum, revealing increased GCP proliferation. Green, BrdU; Blue, DAPI. Scale bars: 100 μm. (**B’**) The number of BrdU^+^ cells per unit size of sagittal sections from the entire cerebellum was quantified in ImageJ (P = 3 × 10 ^−4^). (**C**) Sections of P3.5 wild-type and LKB1^Atoh1^ CKO cerebellum were stained with anti-caspase3 antibody. No significant change in the number of caspase3^+^ cells was observed in the mutant cerebellum compared with the wild-type cerebellum. Green, caspase3; Blue, DAPI. Scale bars: 100 μm. (**C’**) The number of the caspase3^+^ cells per unit size of sagittal sections from the entire cerebellum was quantified in ImageJ. No significant change in the number of caspase3^+^ cells were found in the mutant cerebellum compared with the wild-type cerebellum (P = 0.53). The error bars indicate the SEM. *P < 0.05; **P < 0.01; ***P < 0.001 compared to the WT by Student’s t-test; n = 4 animals for each group.

In our study, the LKB1^Atoh1^ CKO mice exhibited a larger cerebellar volume with more lobules compared to the wild-type mice. Given that cerebellar growth and foliation depend on the proliferation of granule cell precursors, we examined the proliferation of GCPs in the control and LKB1^Atoh1^ CKO cerebellum during early development. Midline sections of the P3.5 cerebellum were labelled with anti-phospho-histone H3 (PH3) antibodies, a marker of mitotic cells, or anti-BrdU antibody to measure GCP proliferation. We observed apparent increases in the numbers of PH3^+^ cells and BrdU^+^ cells in the LKB1^Atoh1^ CKO cerebellum compared with the controls (Fig. 5A,B). The quantification of the relative level of PH3^+^ and BrdU^+^ cells also indicated significant increased proliferation of GCPs in the LKB1^Atoh1^ CKO mice compared with the wild-type mice (Fig. 5A,B). These results suggested that the LKB1 deficiency increased granule cell proliferation precursors in the LKB1^Atoh1^ CKO mice. We next examined the apoptosis in the developing cerebellum at P3.5 mice. By immunostaining, cerebellar cryosections were labeled with an anti-Cleaved Caspase3, an apoptosis marker (Fig. 5C). However, no significant difference of caspase 3^+^ cells was detected in the LKB1^Atoh1^ CKO mice compared with the wild-type mice. The quantification of the relative level of caspase 3^+^ cells also showed no significant change in the LKB1^Atoh1^ CKO mice (Fig. 5C). These results suggested that the deletion of LKB1 did not affect the apoptosis of granule cell precursors in the LKB1^Atoh1^ CKO mice.

### LKB1 deficiency caused defects in granule cell migration in the LKB1^Atoh1^ CKO mice

**Figure 6:**
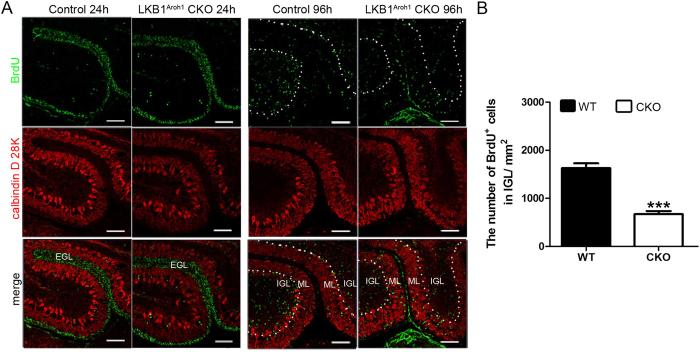
Migration failure of the cerebellar granule cells in the LKB1^Atoh1^ CKO mice. (**A**) BrdU pulse chase experiments at P7. The migration of the GCs in wild-type and LKB1^Atoh1^ CKO cerebella were analyzed at 24 h and 96 h after the BrdU pulse. BrdU immunostaining was counterstained using CalbindinD28K in sagittal sections of the cerebellum. 24 h after the BrdU pulse, the BrdU^+^ cells started departing from the EGL and entered the ML, but had not yet reached the IGL, in both the wild-type and mutant cerebellum. 96 h after the BrdU pulse, significant number of the BrdU^+^ cells were observed in the IGL in the wild-type cerebellum, but much less BrdU^+^ cells were found in the IGL of the mutant cerebellum. (**B**) Quantification of BrdU^+^ cells confirmed that much less BrdU^+^ cells were found in the IGL of mutant cerebellum compared with the wild-type cerebellum. These observations showed that GC migration was disrupted in the LKB1^Atoh1^ CKO cerebellum (P = 2 × 10 ^−4^). The error bars indicate the SEM. *P < 0.05; **P < 0.01; ***P < 0.001 compared to the WT by Student’s t-test; n = 4 animals for each group. Green, BrdU; Red, CalbindinD28K; Blue, DAPI. Scale Bars: 100 μm.

We examined the migration of GCs by BrdU pulse chase experiments to investigate if the GCP migration was disrupted by LKB1 deficiency. Samples were examined at 24 h and 96 h after a BrdU pulse at P7. 24 h after the BrdU pulse, the BrdU-labelled cells were primarily located in the EGL in both the wild type and LKB1^Atoh1^ CKO cerebellum (Fig. 6A). However, 96 h after the BrdU pulse, most of BrdU^+^ cells in the control cerebellum already passed the ML and PCL and reached the IGL (Fig. 6A). In contrast, much less BrdU^+^ cells were observed in the IGL in the LKB1^Atoh1^ CKO mice compared with the wild-type mice (Fig. 6A). The quantitative analysis also showed a significantly decreased number of BrdU^+^ cells in the IGL in the LKB1^Atoh1^ CKO mice compared with the wild-type mice (Fig. 6B). Result indicated a failure of the GC migration in the LKB1^Atoh1^ CKO mice.

### LKB1 inactivation results in ectopic generation of UBCs in the LKB1^Atoh1^ CKO cerebellum

**Figure 7:**
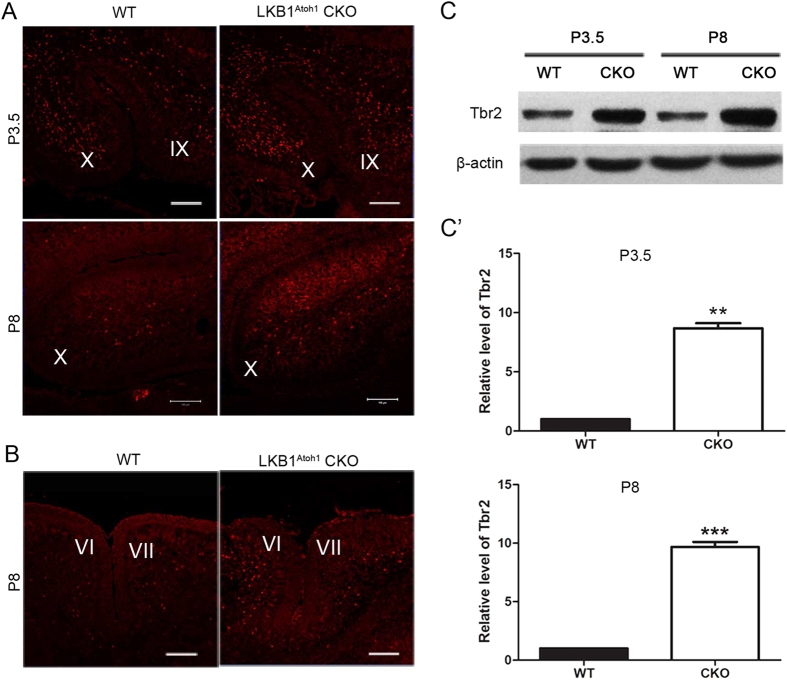
UBC overproduction in the LKB1^Atoh1^ CKO mice. (**A**) Cerebellar sections were stained with an anti-Tbr2 antibody, a marker of UBCs, and imaged in folia IX and X. The P3.5 and P8 LKB1^Atoh1^ CKO cerebellum showed an obvious increase in the number of Tbr2^+^ cells, compared to the controls. Scale Bars: 100 μm. (**B**) The P8 LKB1^Atoh1^ CKO cerebellum showed a large number of Tbr2^+^ cells in folia VI and VII, while only a few Tbr2^+^ cells were observed in the wild-type cerebellum. (**C**) Western blot analysis of the Tbr2 protein in the wild-type and mutant cerebellum. The results showed that the level of the Tbr2 protein was significantly increased in the P3.5 and P8 LKB1^Atoh1^ CKO mice (CKO) compared to the controls (WT). (**C’**) Quantification of the Western blot results for the Tbr2 protein in the wild-type (WT) and LKB1^Atoh1^ CKO cerebellum (CKO). The data were normalized against the results from the wild-type (P_P3.5_ = 2.4 × 10 ^−3^; P_P8_ = 6 × 10 ^−5^). The error bars indicate the SEM. *P < 0.05; **P < 0.01; ***P < 0.001 compared to the WT by Student’s t-test; n = 4 animals for each group.

In our study, we utilized Atoh1-Cre, which specifically targets the granule cell precursors in the developing RL that give rise to both GCs and UBCs^3^,^25^. In addition to GCs, the UBCs are the other known RL-derived interneurons that are enriched in folia IX and X in the adult mice^4^. In the developing cerebellum, the UBCs exit the RL during late embryonic stages and then migrate through the white matter into the IGL until P10^1^,^3^,^4^. Using Tbr2 immunostaining, a marker of UBCs, we found that the UBCs were significantly increased in folia IX and X of the P3.5 and P8 LKB1^Atoh1^ CKO cerebellum compared to the controls (Fig. 7A). Strikingly, we also observed an apparent increase of the UBCs in folia VI and VII of the P8 mutant cerebellum, while the UBCs were rarely identified in this region in the controls (Fig. 7B). We analysed the level of Tbr2 proteins using Western blot analysis. The result showed that the total amount of Tbr2 protein was significantly increased in the P3.5 and P8 LKB1^Atoh1^ CKO mice compared to the wild-type mice (Fig. 7C,C’). These findings suggested that LKB1 deficiency resulted in UBC overproduction during the postnatal development of the mutant cerebellum. This result is consistent with the increased proliferation of the GCPs in the LKB1^Atoh1^ CKO mice.

### LKB1 deficiency increased sonic hedgehog signalling and decreased the level of the pAMPK protein in the LKB1^Atoh1^ CKO cerebellum

**Figure 8:**
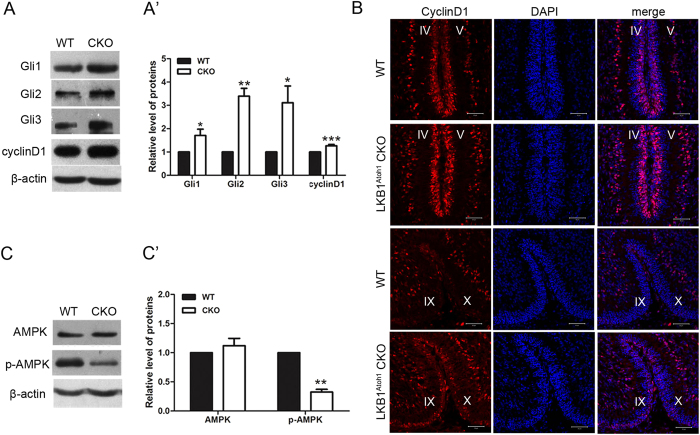
Expression levels of Glis, cyclinD1 and AMPK proteins. (**A**) Western blots of the cerebellum showed that the Gli1, Gli2, Gli3 and cyclinD1 proteins were significantly increased in the P3.5 LKB1^Atoh1^ CKO cerebellum (CKO), compared to the controls (WT). (**A’**) Quantification of the Western blot results for the Gli1, Gli2, Gli3 and cyclinD1 proteins. The data were normalized against the results of the wild-type (P_Gli1_ = 0.03; P_Gli2_ = 0.002; P_Gli3_ = 0.04; P_CyclinD1_ = 4.9 × 10 ^−4^). (**B**) Immunofluorescence of cyclinD1 in P3.5 wild-type and LKB1^Atoh1^ CKO cerebellum. The sections were stained with an anti-cyclinD1 antibody and imaged in folia IV/V and IX/X. The number of cyclinD1^+^ cells in the mutant cerebellum were increased compared to the control. Red, cyclinD1; Blue, DAPI. Scale Bars: 50 μm. (**C**) Western blot analysis of AMPK and phosphorylated AMPK (pAMPK) in the P3.5 cerebellum. There was no obvious change in the total AMPK protein, while the level of pAMPK was significantly reduced in the mutant cerebellum compared to the controls. (**C’**) Quantification of the Western blot results for the AMPK and pAMPK proteins. The data were normalized against the results of the wild-type (P_AMPK_ = 0.3; P_pAMPK_ = 2 × 10 ^−3^). The error bars indicate the SEM. *P < 0.05; **P < 0.01; ***P < 0.001 compared to the WT by Student’s t-test; n > 4 animals for each group.

Foliation of the mouse cerebellum primarily occurs during the first two postnatal weeks and is accompanied by tremendous GCP proliferation^29^. It was reported that the sonic hedgehog signalling (Shh) positively correlates with cerebellar foliation, both spatially and temporally^30^, and that Gli1, Gli2 and Gli3 are the main mediators driving Shh-induced GCP proliferation^31^,^32^,^33^,^34^. To determine whether Shh signalling was altered in the mutant cerebellum, we examined the levels of Gli gene mRNAs and proteins by quantitative real time PCR (qRT-PCR) (data not shown) and Western blotting (Fig. 8A,A’). Both of these results showed that the amount of Gli1, Gli2 and Gli3 was significantly increased in the P3.5 mutant cerebellum compared to the controls (Fig. 8A,A’). These results demonstrated that LKB1 deletion led to a significant increase of Shh signalling in the mutant cerebellum. We also examined whether LKB1 deficiency alters the expression of the Shh signalling target gene cyclinD1. CyclinD1 was a direct target of the Shh pathway and functions to regulate the cell cycle progression of granule cell precursors by increasing G1 cyclin expression in the cerebellum^33^. Western blot data showed that the cyclinD1 expression was significantly increased in the mutant cerebellum compared to the controls (Fig. 8A,A’). CyclinD1 immunostaining of cerebellar sections also showed that the cyclinD1^+^ cells were increased in the mutant cerebellum at P3.5 (Fig. 8B). Thus, the LKB1 deficiency in the LKB1^Atoh1^ CKO cerebellum caused an increase of Shh signalling and cyclinD1, which resulted in the over proliferation of the GCPs during cerebellar development.

**Figure 9:**
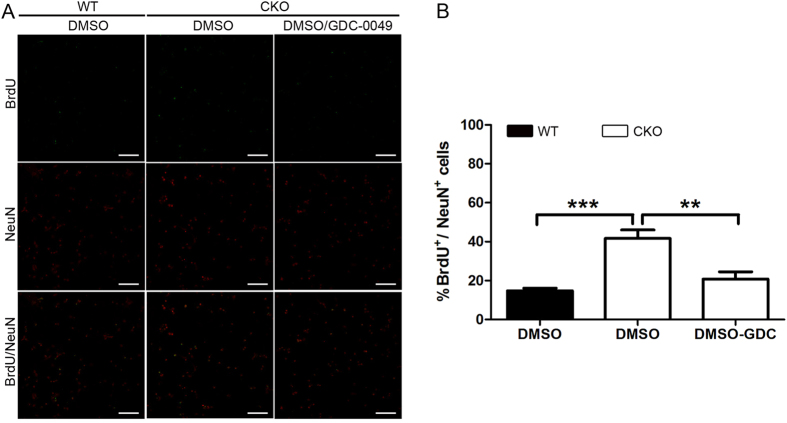
*In vitro* assays for proliferation of GCPs from mutant cerebellum. (**A**) GCPs from P7 cerebellum were cultured and immunostained using anti-NeuN and anti-BrdU antibody. More BrdU^+^ cells among the NeuN^+^ GCs were observed in the mutant GCPs compared with the wild-type GCPs. The rate of the BrdU^+^ cells among NeuN^+^ GCs significantly reduced after treatment with Shh inhibitor GDC-0049. (**B**) Quantitative analysis confirmed the increased BrdU^+^ cells among the NeuN^+^ GCs in the mutant GCPs (P = 3 × 10 ^−6^), whereas Shh inhibitor GDC-0049 reduced the BrdU^+^ cells among the NeuN^+^ GCs. These results indicated that loss of LKB1 increased the Shh-induced proliferation of GCPs (P = 4.2 × 10 ^− 3^). Scale bars: 100 μm. The error bars indicate the SEM. *P < 0.05; **P < 0.01; ***P < 0.001 compared to the WT by Student’s t-test; n > 4 animals for each group.

**Figure 10:**
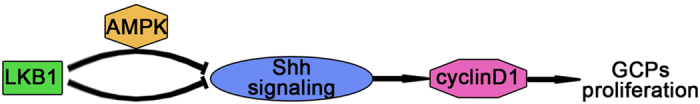
LKB1 function in GCP proliferation during cerebellar development. LKB1 may regulate cerebellar foliation and development by controlling the GCP proliferation via Shh signalling, which controls the G1 cyclins during cerebellar development. In our mutant mice, LKB1 deletion significantly increased the level of Shh signalling, which may increase cyclinD1. These changes resulted in an abnormal increase in GCP proliferation, which caused the cerebellar defects and extra lobules. AMPK likely acts as a mediator between LKB1 and Shh signalling in the LKB1^Atoh1^ CKO cerebellum.

To confirm if LKB1 regulates Shh-induced GCP proliferation in mice, we cultured the GCPs from wild-type and LKB1^Atoh1^ CKO cerebella and analyzed the proliferation of the GCPs by immunostaining with BrdU and NeuN. More BrdU^+^ cells among the NeuN^+^ GCs were observed in the mutant GCPs compared with the wild-type GCPs, indicating increased proliferation of mutant GCPs *in vitro*. In another group of experiments, cultured GCP from mutant mice were treated with Shh inhibitor GDC-0049 and GCP proliferation was examined. We found that the relative level of BrdU^+^ cells among the NeuN^+^ GCs significantly reduced with GDC-0049 treatment compared with those without GDC-0049 (Fig. 9A). The quantitative analysis of the relative levels of BrdU^+^ cells among NeuN^+^ cells also displayed an increased proliferation of cultured GCPs from the mutant cerebellum, and apparent reduction in GCP proliferation by GDC-0049 treatment (Fig. 9B). Thus, these *in vitro* data are consistent with the *in vivo* results suggesting that the LKB1 deficiency caused the increased proliferation of GCPs through Shh pathway (Fig. 10).

We cannot determine whether LKB1 directly affect Shh signalling in the LKB1^Atoh1^ CKO mice. According to previous reports, LKB1 was responsible for activating AMPK^35^,^36^,^37^,^38^, and phosphorylated AMPK negatively correlates with sonic hedgehog signalling^39^. To determine whether AMPK was altered in the mutant cerebellum, we examined the level of the AMPK protein. The Western blot results showed that the total AMPK protein levels were not altered, while phosphorylated AMPK was decreased in the mutant mice compared to the control mice (Fig. 8C,C’). We suggested that AMPK may be an intermediate protein between LKB1 and Shh signalling (Fig. 10).

## Discussion

Previous studies have reported that LKB1 regulates cell proliferation, migration, differentiation and the establishment of cell polarity during mammalian development^13^,^16^,^40^,^41^,^42^,^43^,^44^. As an important serine/threonine kinase, LKB1 plays a key role in multiple developmental signalling pathways and the regulation of life activities. We detected that LKB1 is highly expressed in the mouse cerebellum, and no studies have reported the function of LKB1 during cerebellar development. Because the null allele of LKB1 led to embryonic lethality with multiple developmental abnormalities, particularly in angiogenesis and the nervous system, we could not investigate cerebellar development in conventional LKB1 knockout mice. In our study, we created LKB1 conditional knockout mice (LKB1^Atoh1^ CKO mice) by inactivating the LKB1 in the granular cell precursors of the cerebellum. We analysed the role of LKB1 in the developing cerebellum of the LKB1^Atoh1^ CKO mice.

In contrast to the control mice, the LKB1^Atoh1^ CKO mice have no significant differences in gross morphology, except for the reduced body weight. Behavioral tests indicated that the mutant mice exhibit impaired motor coordination. Starting at approximately P7, the cerebellum displayed larger sizes and more lobules in the mutant mice. The mutant cerebellum also exhibited an overproduction of GCPs and UBCs and impaired GC migration during the cerebellar development. We also found aberrant Purkinje cells development in the LKB1^Atoh1^ CKO mice. These phenotypes indicate that LKB1 is essential for mouse cerebellar development.

### Effects of LKB1 deletion on granule cells precursors

LKB1 is highly expressed in GCPs, indicating its possible important role in GCP development. Granule cells are the most numerous neurons in the cerebellum and are essential to cerebellar foliation and morphogenesis. In our study, we observed obviously larger sizes and more lobules in the cerebella of LKB1^Atoh1^ CKO mice. GCP proliferation was tested and results revealed a hyper-proliferation of GCPs in the mutant cerebellum. In addition, the apoptosis of GCPs was examined to determine whether changes in apoptosis altered the number of GCs. We did not observe more neural progenitor cells that underwent apoptosis in the mutant mice compared with wild-type mice. Therefore, we concluded that the overproduction of GCs was caused by increased GCP proliferation rather than less cell death in the cerebellum of mutant mice.

Appropriate granule cells migration is crucial for proper formation of the laminated structure in the developing cerebellum^10^. Impaired migration usually results in motor dysfunction and ataxia^45^. Genetic mutations and environmental abnormalities may affect the migration of neurons and result in abnormal development^46^,^47^,^48^. During the migration of GCs, starting at P7, the GCs depart the EGL, pass the ML and PCL, and reach the IGL. The maximum rate of cell migration in mice occurs between P7 and P12. Almost all of the progenitors have migrated into the IGL of cerebella at P15 mice^49^. For migration assay of the GCs in cerebelum, BrdU pulse chase experiments were usually used to examine the time course of the migration of cerebellar granule cells in postnatal mice^49^. In our study, most of BrdU^+^ cells in control cerebellum reached the IGL, while a significantly decreased number of BrdU^+^ cells was found in the IGL of the mutant cerebellum 96 h after the BrdU pulse at P7. This result indicated that LKB1 deletion disrupted GC migration to the IGL in the LKB1^Atoh1^ CKO mice. Thus, the loss of LKB1 not only caused increased proliferation of GCPs but also affected GCP migration during the cerebellar development in mice.

### LKB1 regulates cerebellar foliation by controlling GCP proliferation

In the development of cerebellum in mice, the principal fissures initiates in the vermis of the cerebella. The five cardinal lobes can be identified by E18.5^29^,^50^. After birth, the cardinal lobes are gradually divided by additional fissures. Mature cerebella have ten lobes in the vermis in mice. Different mechanisms were proposed regarding cerebellar foliation^29^,^51^, but the precise mechanism remains unclear. Cerebellar growth and foliation depend on GCP proliferation in the developing cerebellum^52^,^53^,^54^,^55^. Expansion in the GCP number is a driving force in the foliation of the cerebellum^29^,^50^,^56^,^57^. In our study, an increased GCP proliferation was detected in the LKB1^Atoh1^ CKO cerebellum. Thus, the expansion of the cerebellar extra lobes probably arose from GCP over-proliferation by LKB1 inactivation in the LKB1^Atoh1^ CKO mice.

Other reports suggest that PCs are also responsible for cerebellar foliation^50^. PCs can anchor the outline of the cortex via their axons projecting to the underlying white matter at positions that define the base of the fissures^50^. In our mutant mice, LKB1^Atoh1^ CKO cerebellum exhibited maldevelopment of PC dendrites, including accumulated and stunted dendrite growth. Given that Atoh1-Cre was not targeted to Purkinje cells in the cerebellum, the disrupted patterning of Purkinje cells may be secondary to LKB1 inactivation in the mutant cerebellum, and it may be caused by abnormal GC development. Abnormal development of granule cells affected the development of PCs^58^,^59^,^60^,^61^,^62^,^63^,^64^. We propose that the increased number of PCs in mutant mice was probably another reason for the malformation of folia.

### LKB1 regulates the proliferation of cerebellar granule cell progenitors via sonic hedgehog signalling

The sonic hedgehog signalling pathway is crucial for the regulation of proliferation, migration and cell differentiation in the developing cerebellum^65^,^66^. During normal cerebellar development, the remarkable expansion of GCPs generates a population of granule cells that can determine the extent of cerebellar foliation during postnatal development^30^,^34^,^55^,^67^,^68^. It was reported that sonic hedgehog (Shh) is responsible for the rapid GCP proliferation^33^,^69^. GCP proliferation was inhibited by the down-regulation of Shh signalling, and elevated Shh signalling could promote GCP proliferation^55^,^70^,^71^,^72^,^73^. Shh signalling is mediated by the Gli family of transcription factors^31^,^32^,^33^,^34^. In the LKB1^Atoh1^ CKO cerebellum, the expression levels of the Gli genes were significant increased, indicating elevated Shh signalling. Cyclin D1 is a direct target of the Shh pathway and functions to regulate cell cycle progression in granule cell precursors by increasing G1 cyclin expression^33^,^74^. The expression of cyclinD1 was significantly increased in the early postnatal LKB1^Atoh1^ CKO cerebellum. The overexpression of cyclinD1 in the mutant cerebellum may promote GCP proliferation. Thus, these results revealed that LKB1 deletion may promote GCP proliferation via elevated Shh signalling and cyclinD1 expression in the developing cerebellum (Fig. 10).

*In vitro* proliferation assays were performed to verify whether LKB1 regulates GCP proliferation through Shh signalling. The proliferation of cultured GCPs from mutant cerebellum significantly increased. GDC-0049, a Shh inhibitor, was then used to block the Shh signalling in the cultured system. GDC-0049 can inhibit SMO, leading to a reduction in GLi1 and Ptch1 expression^75^. Our result indicated that the proliferation of mutant GCPs was significantly reduced in the presence of GDC-0049. Therefore, in consistency with the data from *in vivo* experiment results, our *in vitro* results support the notion that LKB1 may regulate the proliferation of GCPs via sonic hedgehog signalling.

Previous reports demonstrated that AMPK can be phosphorylated by LKB1^36^,^37^,^38^ and can also negatively regulate Shh signalling by interacting with Gli1 *in vitro*^39^. We examined the expression of AMPK by Western blot and found that the mutant cerebellum had similar total AMPK levels and the significantly decreased pAMPK. Thus, AMPK likely acts as an intermediate protein between LKB1 and Shh signalling in the developing cerebellum (Fig. 10).

### LKB1 and tumourigenesis

The previous studies characterizing LKB1 function in mice showed that LKB1 is also considered to be a critical tumour suppressor gene and is mutated in tumours. LKB1 is involved in various types of cancers. Medulloblastoma is the most common malignant cerebellar tumour during cerebellar development. Abnormal developmental processes may result in medulloblastoma tumours, which are frequently found near the surface of the cerebellum^76^. In our study, we generated viable LKB1^Atoh1^ CKO mice and found that loss of LKB1 in the cerebellum led to the abnormalities in GCP proliferation and migration during postnatal neurogenesis. Interestingly, the adult LKB1^Atoh1^ CKO cerebellum exhibited a larger cerebellar volume and size with additional lobules, but there was no tumourigenesis in the cerebellum. Tumourigenesis occurred by cells undergoing uncontrolled cell division. Although the loss of LKB1 caused over-proliferation of GCPs in the LKB1^Atoh1^ CKO cerebellum, we speculate that ablation of LKB1 alone may not be sufficient to cause cancer. Although LKB1 deficiency did not promote tumourigenesis in the cerebellum, LKB1^Atoh1^ CKO mice can provide an ideal mouse model to study the function of LKB1 gene in cerebellar development and motor dysfunction throughout cerebellar neurogenesis.

## Additional Information

**How to cite this article**: Men, Y. *et al.* LKB1 Regulates Cerebellar Development by Controlling Sonic Hedgehog-mediated Granule Cell Precursor Proliferation and Granule Cell Migration. *Sci. Rep.* **5**, 16232; doi: 10.1038/srep16232 (2015).
